# Successful Second Awake Craniotomy Reoperation With Dexmedetomidine After an Initial Abandonment Case Due to Restlessness

**DOI:** 10.7759/cureus.61506

**Published:** 2024-06-01

**Authors:** Yutaro Ikki, Takehito Sato, Kimitoshi Nishiwaki

**Affiliations:** 1 Anesthesiology, Handa City Hospital, Handa, JPN; 2 Anesthesiology, Nagoya University Hospital, Nagoya, JPN; 3 Anesthesiology, Nagoya University Graduate School of Medicine, Nagoya, JPN

**Keywords:** restlessness, asleep-awake-awake methods, propofol, dexmedetomidine, awake craniotomy

## Abstract

Awake craniotomy (AC) is sometimes aborted due to poor arousal and restlessness. Dexmedetomidine (DEX), an α2-adrenoreceptor agonist, has sedative, analgesic, and anesthetic-sparing effects, with a low risk of respiratory depression, making it effective for intraoperative pain and agitation during the awake phase. We report a case in which AC was successfully performed in combination with low-dose continuous administration of DEX during reoperation in a patient who experienced poor arousal and restlessness during their first surgery, leading to the abandonment of AC. The patient is a 48-year-old male who is scheduled for AC reoperation. Two years ago, the first AC was scheduled and performed under anesthesia with propofol and remifentanil. However, AC was abandoned due to poor intraoperative arousal and restlessness. At reoperation, general anesthesia was induced with propofol and continuous administration of remifentanil (0.1 µg/kg/min); following anesthesia induction (continuous infusion of propofol, remifentanil, and a bolus infusion of fentanyl), DEX was also administered (0.2 µg/kg/hour). We performed a scalp nerve block. Before the awake phase, the propofol dose was decreased as was DEX to 0.1 µg/kg/hour, and propofol and remifentanil were discontinued. The patient gradually awoke without any agitation and restlessness 24 min after stopping propofol and remifentanil and could perform language tasks without any complications. In this case, AC was successfully performed in combination with continuous low-dose administration of DEX at the time of reoperation in a patient who experienced poor arousal and restlessness during their first operation and had to discontinue AC.

## Introduction

Awake craniotomy (AC) minimizes the risk of complications and enables the proper resection of lesions while evaluating patient symptoms. However, in some cases, it is necessary to abandon AC because of poor arousal and restlessness in the patient. According to a previous report [1], the failure rate of AC was reported at 6.4%, and the main cause of failure was the lack of intraoperative communication.

Dexmedetomidine (DEX) is an α2-adrenoreceptor agonist with sedative, analgesic, and anesthetic-sparing effects [2,3]. Due to the low risk of respiratory depression and effectiveness for intraoperative pain and agitation during the awake phase, DEX is frequently used, especially in the monitored anesthetic care (MAC) method of AC [4].

Herein, we report that AC was successfully performed in combination with low-dose continuous administration of DEX during reoperation in a patient who experienced poor arousal and restlessness during their first surgery and had to abandon AC.

## Case presentation

Written informed consent for case reports and DEX off-label use were obtained from the patient. This study was also approved by the Institutional Review Board at Nagoya University Hospital (approval number: 2023-0459).

The patient was a 48-year-old male (height: 179 cm; weight: 82 kg; BMI: 25.6 kg/m^2^) who was diagnosed with a left temporal lobe tumor located in an eloquent area and scheduled to undergo AC for tumor resection.

The patient did not receive any premedication. General anesthesia was induced with propofol and continuous administration of remifentanil (0.1 µg/kg/min) and bolus doses of fentanyl (150 µg). The effect-site concentration of propofol was set at 4.5 µg/mL. After confirming the loss of consciousness, a supraglottic airway device (Igel # 4 Intersurgical, Wokingham, Berkshire, UK) was inserted, and mechanical ventilation was initiated. Vital signs were recorded using non-invasive and invasive blood pressure monitoring, capnography, electrocardiography, and pulse oximetry (SpO_2_). After inserting the arterial and urinary catheters, we performed scalp blocks (supraorbital and supratrochlear, greater and lesser occipital, auriculotemporal, and zygomaticotemporal branch nerves) with 0.375% ropivacaine and 1:200,000 adrenaline to reduce intraoperative pain. During anesthetic maintenance, we gradually decreased the continuous dose of propofol as much as possible, targeting a bispectral index (BIS) value of 40-60. The target-controlled infusion (TCI) of propofol was 4.0 µg/mL.

Levetiracetam (500 mg) was administered as an antiepileptic, a low dose of dexamethasone (6.6 mg) was administered as an antiemetic, and mannitol (150 mL) was administered to decrease the intracranial pressure before the awake phase.

After the dura was opened, propofol and remifentanil administration were halted. After 24 min, he began to breathe spontaneously but was unable to regain full consciousness and exhibited uncontrollable movement and difficulty responding. For safety, he was re-sedated 43 min after halting propofol administration, whereby we attempted to wake up the patient several times unsuccessfully. The Richmond agitation-sedation scale score was +3. Finally, we abandoned AC and performed tumor resection under general anesthesia.

Two years later, due to the re-enlargement of the tumor, AC was requested again. Based on a discussion with the neurosurgeon, we have decided to use general anesthesia with a low dose of DEX in order to prevent restlessness during the awake phase for reoperation.

**Figure 1:**
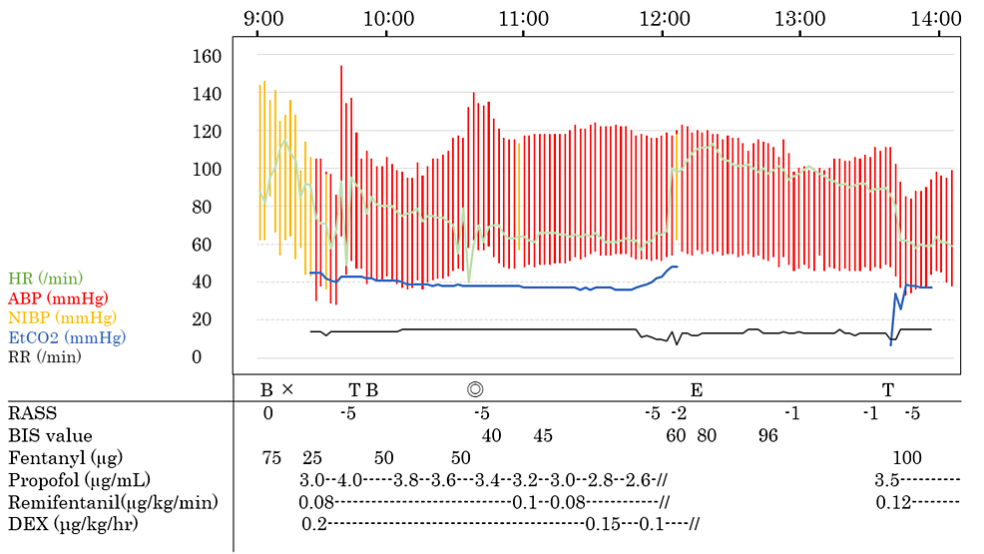
Anesthesia record of reoperation. X, start of anesthesia time; ◎, start of the operation; HR, heart rate; ABP, arterial blood pressure; NIBP, non-invasive blood pressure; EtCO_2_, end-tidal carbon dioxide; RR, respiratory rate; DEX, dexmedetomidine; RASS, Richmond agitation-sedation scale; BIS, bispectral index; B, regional nerve block; T, intubation; E, extubation

Anesthesia was induced with a continuous infusion of propofol (effect-site concentration was 3.0 µg/mL), remifentanil (0.08 µg/kg/min), and a bolus infusion of fentanyl (100 µg). In addition to propofol and remifentanil, DEX was administered (0.2 µg/kg/hour). After confirmed loss of consciousness, an Igel®, artery catheter, and a urinary catheter were inserted. We started mechanical ventilation, targeting normocapnia. We performed a scalp nerve block in the same manner as in the previous operation. Before the awake phase, the TCI of propofol was decreased to 2.6 µg/mL. DEX was also gradually decreased to 0.1 µg/kg/hour, targeting a BIS value of 40-60. Upon arousal, propofol and remifentanil were discontinued; however, DEX was continued at 0.1 µg/kg/hour. The patient gradually awoke 24 min after stopping propofol without any agitation and restlessness. The patient complained of discomfort caused by the urinary catheter; therefore, it was removed. After the urinary catheter was removed, the patient could perform intraoperative language tasks (the Richmond agitation-sedation scale score was -2 at the awake phase), and DEX administration was discontinued after tumor resection was initiated (Figure 1). He was able to perform language tasks without restlessness and agitation during the awake phase. After 84 min of the awake phase, general anesthesia was induced with propofol and remifentanil again, and AC was completed without any complications such as high intracranial pressure, mass bleeding, and difficulty in dural suturing.

## Discussion

This report details a case of AC that was successfully performed in combination with low-dose continuous administration of DEX at the time of reoperation in a patient who experienced arousal and restlessness during the first surgery, leading to the abandonment of AC. Poor arousal in AC has been reported to result from residual anesthetic effects [5]. In Japan, the frequency of poor arousal was reportedly over 10% in patients who could not perform intraoperative tasks [6]. Restlessness and agitation can also be caused by pain, mental tension, nausea, and residual anesthetic effects [6]; however, the reason for restlessness in AC has not been fully elucidated.

DEX is frequently administered during AC, mainly in the MAC method, and its effectiveness has been demonstrated [7,8]. In Japan, AC is primarily performed using the asleep-awake-asleep (AAA) method. The use of DEX in the AAA method reduces restlessness, agitation, and pain during the awake phase [9]. Although its use under general anesthesia is off-label in Japan, there have been reports of its use in elderly patients with AC under general anesthesia [10]. Based on previous reports [10,11], a low dose of DEX was administered continuously without loading or bolus administration to prevent hypotension, severe bradycardia, and excessive sedation. As mentioned above, DEX is used clinically for perioperative high-quality sedation, anesthesia, and analgesia. On the other hand, DEX has also bad aspects. Firstly, the context-sensitive half-time of DEX is longer than propofol [12,13]. Thus, there are some risks of excessive sedation. At the awake phase of reoperation, the patient woke up without restlessness while DEX was infused. After we recognized that he was able to perform neurological tasks, we stopped DEX because the context-sensitive half-time of DEX was longer than propofol and there was a possibility of preventing arousal. Secondly, it is said that intraoperative seizures detected with electrocorticography during AC occurred more frequently in patients receiving DEX than propofol in a recent report [14]. However, in our case, we used both DEX and propofol. Using them together might prevent intraoperative seizures from occurring because the dose of DEX was small and propofol had an anticonvulsant effect.

The potential cause of poor arousal and restlessness in the first operation was unclear, but it assumed that the propofol dose (effect-site concentration of 4.5 µg/mL) was higher than that in the second operation (effect-site concentration of 3.0 µg/mL), and the patient’s perceived discomfort from the urinary catheter in the second surgery was strong upon the awake phase. In our experience, catheter-related bladder discomfort frequently occurred in men during the awakening phase. It has also been reported that intraoperative DEX administration reduced the incidence and severity of postoperative urinary catheter-related bladder discomfort [15,16]. Therefore, the introduction of his DEX and removal of the urinary catheter during the secondary surgery may have reduced his discomfort and pain. To the best of our knowledge, there have been no reports on the use of DEX in patients with a history of AC failure due to poor arousal. In this case, general anesthesia was administered in combination with DEX, making it possible to perform AC, and appropriate sedation and analgesia were achieved during the awake phase.

## Conclusions

In conclusion, we report a case in which AC was successfully performed in combination with continuous low-dose administration of DEX at the time of reoperation in a patient who experienced poor arousal and restlessness during their first operation and had to discontinue AC. General anesthesia was administered in combination with DEX, making it possible to perform AC, and appropriate sedation and analgesia were achieved during the awake phase.
